# *In Vitro* Activity of MRX-8 and Comparators Against Clinical Isolated Gram-Negative Bacilli in China

**DOI:** 10.3389/fcimb.2022.829592

**Published:** 2022-05-12

**Authors:** Shi Wu, Dandan Yin, Peiyuan Zhi, Yan Guo, Yang Yang, Demei Zhu, Fupin Hu

**Affiliations:** ^1^ Institute of Antibiotics, Huashan Hospital, Fudan University, Shanghai, China; ^2^ Key Laboratory of Clinical Pharmacology of Antibiotics, Ministry of Health, Shanghai, China

**Keywords:** MRX-8, minimal inhibitory concentration, colistin, polymyxin B, gram-negative bacteria

## Abstract

To evaluate *in vitro* antibacterial activity of MRX-8 against gram-negative bacteria recently isolated from China, 765 clinical isolates were collected randomly from 2017 to 2020, including *Enterobacterales* and *P. aeruginosa* and *A. baumannii*, *S. maltophilia, B. cepacia, Alcaligenes app.* and *Haemophilus* spp. isolates. All strains were performed with antimicrobial susceptibility testing by broth microdilution method according to the CLSI 2021. Antimicrobial agents included MRX-8, polymyxin B, colistin, amikacin, ceftriaxone, ceftazidime, cefepime, ceftazidime-avibactam, cefoperazone-sulbactam, meropenem, ciprofloxacin, ampicillin, ampicillin-sulbactam and levofloxacin. For carbapenem-susceptible and carbapenem-resistant *E.coli* isolates, the MIC_50/90_ of MRX-8 was 0.125/0.25 mg/L and 0.06/0.125 mg/L, respectively. For carbapenem-susceptible and carbapenem-resistant *K. pneumoniae* isolates, the MIC_50/90_ of MRX-8 was 0.25/0.5 mg/L and 0.125/0.5 mg/L, respectively. For polymyxins (polymyxin B and colistin)-resistant *E. coli* and *K. pneumoniae*, MIC_50_ of MRX-8 was 4-16 mg/L and MIC_90_ was >32 mg/L. The MIC_50_ and MIC_90_ of MRX-8 for other *Klebsiella* spp. except *K. pneumoniae*, *Citrobacter* spp., *S. enterica* and *Shigella* spp. isolates ranged 0.06-0.125 mg/L and 0.06-0.25mg/L, respectively. For *Morganella* spp.*, Proteus* spp.*, Providencia* spp.*, Serratia* spp., *S. maltophilia* and *B. cepacia*, all MIC_50_ of MRX-8 was >32mg/L. For carbapenem susceptible and resistant *P. aeruginosa*, the MIC_50_ and MIC_90_ of MRX-8 was both 1mg/L, and that for *A. baumannii* was 0.5mg/L and 0.5-1mg/L. For *Alcaligenes* spp. and *Haemophilus* spp., MIC_50/90_ was 1/4 mg/L and 0.25/0.5 mg/L. MRX-8 was more effective against most clinically isolated gram-negative isolates, including carbapenem-resistant *E. coli*, *K. pneumoniae*, *P. aeruginosa* and *A. baumannii*, highlighting its potential as valuable therapeutics.

## Introduction

In recent years, antimicrobial resistance has become a serious public health problem. With the rapid dissemination of multidrug-resistant gram-negative bacteria especially for carbapenem-resistant Gram-nagetive bacilli, the selection of antimicrobial agents for treating infections caused by these strains is limited. According to the results from China Antimicrobial Surveillance Network (CHINET) from 2005 to 2020, the drug resistance rates of *Klebsiella pneumoniae* to imipenem and meropenem were from 3.0% to 23.2% and from 2.9% to 24.2%, respectively. In 2020, the drug resistance rates of *Pseudomonas aeruginosa* to imipenem and meropenem were 23.2% and 19.3%, respectively; the drug resistance rates of *Acinetobacter baumannii* to imipenem and meropenem were more than 70% (https://www.chinets.com).

Polymyxin B (PMB) and colistin, called polymyxins, are two of the few drugs available for selection, showing high antibacterial activity against common clinically isolated Gram-negative bacilli. As the CLSI described, colistin and polymyxin B are considered equivalent agents, so MICs obtained from testing colistin predict MICs to polymyxin B and vice versa (Wayne, 2021). Polymyxins were first discovered in the 1940s. It is a group of cyclic peptide antibiotics with A~E components isolated from the Gram-positive spore-forming bacterium *Paenibacillus polymyxa* (Nang et al., 2021). However, it was soon abandoned in clinical practice due to its high renal toxicity, and has since been mainly used in veterinary drugs and feed additives (Vaara, 2019). With the emergence of carbapenem-resistant Gram-negative bacilli, polymyxins were used in clinical practice as one of the “last line of defense” against multi-resistant gram-negative bacterial infections (Bergen et al., 2012; Brown and Dawson, 2017; Tsuji et al., 2019). MRX-8 is a novel polymyxin analogue in development for the treatment of infections caused by multi-drug resistant Gram-negative pathogens, including *E. coli, K. pneumoniae, P. aeruginosa* and *A. baumannii*, and promising *in vivo* activity was noted for MRX-8 against those pathogens in the mouse thigh and lung model (Lepak et al., 2020). Compared with Polymyxin B, MRX-8 was developed using a “soft drug design,” which represents a new approach aimed at designing safer drugs with an increased therapeutic index by integrating metabolism and detoxification factors into the drug design process (Lepak et al., 2020; Duncan, 2022). Based on the results, apply of MRX-8 in clinical for drug-resistant pathogens in near future are expected and more preclinical evaluation of MRX-8 are needed. In this study, we evaluated the antibacterial activity of MRX-8 and comparators against clinically isolated gram-negative bacteria in China.

## Materials and Methods

### Bacterial Strains

A total of 765 nonduplicate clinical isolates were collected randomly from 52 hospitals in 20 provinces and cities and 3 autonomous regions including Beijing, Shanghai, Zhejiang, Guangdong and Inner Mongolia from 2017 to 2020, including *E. coli* (n=122, 18 of polymyxins-resistant strains, 58 of polymyxins-susceptible and carbapenem-susceptible strains, 46 of polymyxins-susceptible and carbapenem-resistant strains), *K. pneumoniae* (n=138, 32 of polymyxins-resistant strains, 46 of polymyxins-susceptible and carbapenem-susceptible strains, 60 of polymyxins-susceptible and carbapenem-resistant strains), *Klebsiella* spp.(n=25), *Citrobacter* spp. (n=25), *Morganella* spp. (n=25), *Proteus* spp. (n=25), *Serratia Spp.* (n=25), *Providencia* spp. (n=25), *Salmonella enterica* (n=17), *Shigella* spp. (n=20), *P. aeruginosa* (n=100, 46 of carbapenem-susceptible strains and 54 of carbapenem-resistant strains), *A. baumannii* (n=113, 43 of carbapenem-susceptible strains and 70 of carbapenem-resistant strains), *S. maltophilia* (n=30), *B. cepacia* (n=30), *Alcaligenes* spp. (n=20) and *Haemophilus* spp.(n=25). Species identification was performed using the matrix-assisted laser desorption ionization–time-of-flight mass spectrometry (Vitek MS; bioMérieux). All strains were mainly isolated from respiratory tract (500/765, 65.4%), blood (38/765, 5.0%), urine (227/765, 29.7%).

### Antimicrobial Susceptibility Testing

MICs were determined by the reference broth microdilution method according to the 31^st^ Clinical and Laboratory Standards Institute (CLSI) (Wayne, 2021). MRX-8, polymyxin B, colistin, amikacin, ceftriaxone, ceftazidime, cefepime, ceftazidime-avibactam, cefoperazone-sulbactam, meropenem, ciprofloxacin, ampicillin, ampicillin-sulbactam and levofloxacin were tested using a dried customized commercially prepared microdilution panel (Sensititre; Thermo Fisher Scientific). And MRX-8 was obtained from the MicuRx company. *E. coli* ATCC 25922, *P. aeruginosa* ATCC 27853, *Haemophilus influenzae* ATCC 49766 and *Haemophilus influenzae* ATCC 49247 were used as the quality control strains in the antimicrobial susceptibility testing. Quality control and interpretation of the results were based on 2021 CLSI breakpoints for all the antimicrobial agents with the exception of polymyxin B and colistin. polymyxin B and colistin MICs were interpreted using the European Committee on Antimicrobial Susceptibility Testing (EUCAST) (Susceptible, MIC ≤ 2 mg/L; Resistant: MIC≥4 mg/L). MRX-8 MICs were interpreted using CLSI breakpoints for polymyxin B or colistin for comparison purpose only.

### Carbapenem-Resistant *Enterobacterales* Definition

As defined by the Centers for Disease Control and Prevention (CDC), the *Enterobacterales* isolates resistant to at least one of the carbapenems (ertapenem, meropenem, doripenem, or imipenem) or produce a carbapenemase were carbapenem-resistant *Enterobacterales* (CRE) (https://www.cdc.gov/hai/organisms/cre/technical-info.html#Definition).

## Results

### *In Vitro* Activity of MRX-8 Against Clinical Isolates

MRX-8 exhibited potent antibacterial activity against both carbapenem-susceptible and carbapenem-resistant *Enterobacterales.* For polymyxins (polymyxin B and colistin)-susceptible *E. coli*, the MIC_50_ of MRX-8 was 0.125mg/L and 0.06mg/L, and the MIC_90_ was 0.25mg/L and 0.125mg/L, when against carbapenem-susceptible and carbapenem-resistant strains, respectively. For polymyxins-susceptible *K. pneumoniae*, carbapenem-susceptible and carbapenem-resistant strains, the MIC_50_ of MRX-8 was 0.25 mg/L and 0.125 mg/L, respectively, and the MIC_90_ was both 0.5mg/L. As expected, MRX-8 also showed poor antibacterial activity against polymyxins-resistant *E. coli* and *K. pneumoniae* with MIC_50_ of 4 mg/L and 16 mg/L, respectively, and MIC_90_ of >32 mg/L (**Tables 1**, **2**). The MIC_50_ and MIC_90_ of MRX-8 for other *Klebsiella* spp., *Citrobacter* spp., *Salmonella enterica* and *Shigella* spp. strains ranged from 0.06 to 0.125 mg/L and 0.06 to 0.25mg/L, respectively. MRX-8 exhibited potent antibacterial activity against both carbapenem-susceptible and carbapenem-resistant *P. aeruginosa* and *A. baumannii*. The MIC_50_ and MIC_90_ of MRX-8 for *P. aeruginosa* was both 1mg/L, and that for *A. baumannii* was 0.5mg/L and 0.5-1mg/L, respectively. MRX-8 showed no antibacterial activity against *Morganella* spp.*, Proteus* spp.*, Providencia* spp.*, and Serratia* spp.*, S. maltophilia* and *B. cepacia* with MIC_50_ of >32 mg/L (**Tables 3**–**5**). MRX-8 exhibited potent antibacterial activity for *Alcaligenes* spp. with MIC_50_ and MIC_90_ of 1mg/L and 4mg/L, respectively (**Table 6**). MRX-8 also exhibited potent antibacterial activity for *Haemophilus* spp. with MIC_50_ and MIC_90_ of 0.25mg/L and 0.5mg/L, respectively (**Table 7**).

**Table 1:** *In vitro* activity of MRX-8 and other comparator agents against 122 of *Escherichia coli* (mg/L).

Isolates	Antimicorbial agents	MIC (mg/liter)			R, %	S, %
		Range	50%	90%		
polymyxin non-resistant and carbapenem-susceptible *Escherichia coli* (n = 58)	MRX-8	0.06-0.5	0.125	0.25	0	100
	Polymyxin B	0.125-1	0.25	0.5	0	100
	Colistin	0.125-1	0.25	0.5	0	100
	Amikacin	1->64	4	8	3.4	93.1
	Ceftriaxone	≤0.06->64	>64	>64	72.4	27.6
	Ceftazidime	0.125->64	8	>64	44.8	41.4
	Cefepime	≤0.06->64	16	>64	67.2	29.3
	Ceftazidime-avibactam	≤0.06->64	0.25	2	8.6	91.4
	Cefoperazone-sulbactam	0.25->64	8	64	17.2	69
	Meropenem	≤0.06-1	0.06	0.125	0	100
	Ciprofloxacin	≤0.06->64	16	>64	70.7	17.2
polymyxin non-resistant and carbapenem-resistant *Escherichia coli* (n = 46)	MRX-8	0.06-0.5	0.06	0.125	0	100
	Polymyxin B	0.125-1	0.25	0.25	0	100
	Colistin	0.125-1	0.125	0.25	0	100
	Amikacin	1->64	8	>64	39.1	56.5
	Ceftriaxone	>64->64	>64	>64	100	0
	Ceftazidime	32->64	>64	>64	100	0
	Cefepime	>64->64	>64	>64	100	0
	Ceftazidime-avibactam	≤0.06->64	>64	>64	84.8	15.2
	Cefoperazone-sulbactam	32->64	>64	>64	97.8	0
	Meropenem	4->16	>16	>16	100	0
	Ciprofloxacin	1->64	>64	>64	100	0
Polymyxin-resistant *Escherichia coli* (n = 18)	MRX-8	2->32	4	>32	94.4	5.6
	Polymyxin B	4->32	4	>32	100	0
	Colistin	4->8	4	>8	100	0
	Amikacin	1->64	2	>64	27.8	66.7
	Ceftriaxone	≤0.06->64	>64	>64	77.8	22.2
	Ceftazidime	0.125->64	64	>64	66.7	33.3
	Cefepime	≤0.06->64	64	>64	66.7	22.2
	Ceftazidime-avibactam	≤0.06->64	0.5	>64	22.2	77.8
	Cefoperazone-sulbactam	0.5->64	32	>64	44.4	38.9
	Meropenem	≤0.06->16	0.06	>16	22.2	77.8
	Ciprofloxacin	0.5->64	32	>64	88.9	5.6

R, resistant; S, susceptible. All the intermediate data were not showed in table.

**Table 2:** *In vitro* activity of MRX-8 and other comparator agents against 138 of *Klebsiella pneumoniae* (mg/L).

Isolates	Antimicorbial agents	MIC (mg/liter)			R, %	S, %
		Range	50%	90%		
polymyxin non-resistant and carbapenem-susceptible *Klebsiella pneumoniae* (n = 46)	MRX-8	0.06-0.5	0.25	0.5	0	100
	Polymyxin B	0.25-1	0.25	0.5	0	100
	Colistin	0.25-1	0.25	0.5	0	100
	Amikacin	0.5-8	1	2	0	100
	Ceftriaxone	0.06->64	0.06	>64	19.6	80.4
	Ceftazidime	0.125-64	0.5	8	8.7	82.6
	Cefepime	0.06-64	0.125	8	17.4	80.4
	Ceftazidime-avibactam	0.06-0.5	0.25	0.5	0	100
	Cefoperazone-sulbactam	0.125-64	0.5	32	2.2	89.1
	Meropenem	0.06-0.06	0.06	0.06	0	100
	Ciprofloxacin	0.06->64	0.125	32	28.3	69.6
polymyxin non-resistant and carbapenem-resistant *Klebsiella pneumoniae* (n = 60)	MRX-8	0.06-2	0.125	0.5	0	100
	Polymyxin B	0.125-2	0.25	1	0	100
	Colistin	0.125-1	0.25	0.5	0	100
	Amikacin	0.25->64	>64	>64	76.7	23.3
	Ceftriaxone	>64->64	>64	>64	100	0
	Ceftazidime	64->64	>64	>64	100	0
	Cefepime	32->64	>64	>64	100	0
	Ceftazidime-avibactam	1->64	2	>64	21.7	76.7
	Cefoperazone-sulbactam	>64->64	>64	>64	100	0
	Meropenem	32-32	32	32	100	0
	Ciprofloxacin	≤0.06->64	64	>64	98.3	1.7
Polymyxin-resistant *Klebsiella pneumoniae* (n = 32)	MRX-8	2->32	16	>32	91.6	9.4
	Polymyxin B	4->32	16	>32	100	0
	Colistin	8->8	>8	>8	100	0
	Amikacin	1->64	>64	>64	78.1	21.9
	Ceftriaxone	0.125->64	>64	>64	96.9	3.1
	Ceftazidime	0.5->64	>64	>64	93.8	6.2
	Cefepime	0.125->64	>64	>64	93.8	6.2
	Ceftazidime-avibactam	0.25->64	2	4	6.2	93.8
	Cefoperazone-sulbactam	1->64	>64	>64	84.4	3.1
	Meropenem	≤0.06->16	>16	>16	78.1	21.9
	Ciprofloxacin	≤0.06->64	>64	>64	87.5	12.5

R, resistant; S, susceptible. All the intermediate data were not showed in table.

**Table 3:** *In vitro* activity of MRX-8 and other comparator agents against 187 of other Enterobacterales (mg/L).

Isolates	Antimicorbial agents	MIC (mg/liter)			R, %	S, %
		Range	50%	90%		
Other *Klebsiella spp.*(n = 25)	MRX-8	0.06->32	0.125	0.125	4	96
	Polymyxin B	0.125->32	0.25	0.5	4	96
	Colistin	0.125->8	0.25	0.5	4	96
	Amikacin	0.5-2	1	2	0	100
	Ceftriaxone	≤0.06->64	0.25	>64	28	72
	Ceftazidime	0.125->64	0.25	>64	24	76
	Cefepime	≤0.06->64	≤0.06	64	20	80
	Ceftazidime-avibactam	≤0.06->64	0.125	>64	16	84
	Cefoperazone-sulbactam	0.125->64	1	>64	16	84
	Meropenem	≤0.06-32	≤0.06	16	12	84
	Ciprofloxacin	≤0.06-4	≤0.06	1	16	80
*Citrobacter spp.* (n = 25)	MRX-8	0.03->32	0.125	0.25	8	92
	Polymyxin B	0.25->32	0.5	1	8	92
	Colistin	0.25->8	0.5	1	8	92
	Amikacin	0.5-16	1	2	0	100
	Ceftriaxone	≤0.06->64	1	>64	44	56
	Ceftazidime	0.125->64	1	>64	44	52
	Cefepime	≤0.06->64	0.06	64	24	64
	Ceftazidime-avibactam	≤0.06->64	0.25	>64	16	84
	Cefoperazone-sulbactam	≤0.06->64	1	>64	28	64
	Meropenem	≤0.06->16	0.06	>16	16	84
	Ciprofloxacin	≤0.06-32	0.06	16	32	68
*Morganella spp.* (n = 25)	MRX-8	32->32	>32	>32	100	0
	Polymyxin B	>32->32	>32	>32	100	0
	Colistin	>8->8	>8	>8	100	0
	Amikacin	1-16	4	8	0	100
	Ceftriaxone	≤0.06->64	1	32	36	52
	Ceftazidime	≤0.06->64	2	64	24	72
	Cefepime	≤0.06->64	0.06	8	12	84
	Ceftazidime-avibactam	≤0.06-1	0.06	0.25	0	100
	Cefoperazone-sulbactam	0.5-16	2	16	0	100
	Meropenem	≤0.06-0.25	0.125	0.25	0	100
	Ciprofloxacin	≤0.06->64	0.125	16	40	60
*Proteus spp.* (n = 25)	MRX-8	>32->32	>32	>32	100	0
	Polymyxin B	>32->32	>32	>32	100	0
	Colistin	>8->8	>8	>8	100	0
	Amikacin	1-32	4	32	0	88
	Ceftriaxone	≤0.06->64	4	>64	64	32
	Ceftazidime	≤0.06-32	0.125	4	4	96
	Cefepime	≤0.06->64	0.125	64	32	60
	Ceftazidime-avibactam	≤0.06-0.25	≤0.06	0.125	0	100
	Cefoperazone-sulbactam	0.5-8	2	8	0	100
	Meropenem	≤0.06-1	0.125	0.25	0	100
	Ciprofloxacin	≤0.06-64	4	64	52	40
*Providencia spp.* (n = 25)	MRX-8	>64->64	>64	>64	100	0
	Polymyxin B	>64->64	>64	>64	100	0
	Colistin	>8->8	>8	>8	100	0
	Amikacin	0.25->64	2	16	8	92
	Ceftriaxone	≤0.06->64	0.5	>64	36	64
	Ceftazidime	≤0.06->64	2	>64	40	56
	Cefepime	≤0.06->64	0.5	>64	40	52
	Ceftazidime-avibactam	≤0.06->64	0.125	>64	20	80
	Cefoperazone-sulbactam	0.125->64	4	>64	20	76
	Meropenem	≤0.06-32	0.125	16	16	80
	Ciprofloxacin	≤0.06-32	4	16	52	36
*Serratia Spp.* (n = 25)	MRX-8	>32->32	>32	>32	100	0
	Polymyxin B	>32->32	>32	>32	100	0
	Colistin	>8->8	>8	>8	100	0
	Amikacin	1-8	2	4	0	100
	Ceftriaxone	0.25->64	0.5	64	24	76
	Ceftazidime	0.125-16	0.5	4	4	92
	Cefepime	≤0.06->64	0.25	>64	16	80
	Ceftazidime-avibactam	≤0.06-1	0.25	1	0	100
	Cefoperazone-sulbactam	0.5->64	4	64	12	80
	Meropenem	≤0.06->16	≤0.06	>16	12	88
	Ciprofloxacin	≤0.06-8	0.125	8	24	76
*Salmonella spp.* (n = 17)	MRX-8	0.06-4	0.25	0.25	5.9	94.1
	Polymyxin B	0.125-4	0.25	0.5	5.9	94.1
	Colistin	0.125-4	0.5	1	5.9	94.1
	Amikacin	0.25-4	1	2	0	100
	Ceftriaxone	≤0.06->64	0.125	32	17.6	82.4
	Ceftazidime	0.125->64	0.5	64	17.6	82.4
	Cefepime	≤0.06-32	0.125	1	5.9	94.1
	Ceftazidime-avibactam	≤0.06-1	0.25	0.5	0	100
	Cefoperazone-sulbactam	0.5-32	1	32	0	88.2
	Meropenem	≤0.06-0.125	0.06	0.06	0	100
	Ciprofloxacin	≤0.06-1	0.25	1	23.5	0
*Shigella spp.* (n = 20)	MRX-8	0.03-0.125	0.06	0.06	0	100
	Polymyxin B	0.125-0.25	0.25	0.25	0	100
	Colistin	0.125-0.25	0.25	0.25	0	100
	Amikacin	2-4	2	4	0	100
	Ceftriaxone	≤0.06->64	0.06	128	40	60
	Ceftazidime	≤0.06-32	0.25	8	10	85
	Cefepime	≤0.06-64	0.5	16	40	60
	Ceftazidime-avibactam	≤0.06-0.25	≤0.06	0.125	0	100
	Cefoperazone-sulbactam	0.25-32	2	16	0	90
	Meropenem	≤0.06 -≤0.06	≤0.06	≤0.06	0	100
	Ciprofloxacin	0.125-16	0.25	16	50	50

R, resistant; S, susceptible. All the intermediate data were not showed in table.

**Table 4:** *In vitro* activity of MRX-8 and other comparator agents against 100 of *Pseudomonas aeruginosa* (mg/L).

Isolates	Antimicorbial agents	MIC (mg/liter)			R, %	S, %
		Rang	50%	90%		
carbapenem-susceptible *Pseudomonas aeruginosa* (n = 46)	MRX-8	0.125-2	1	1	0	100
	Polymyxin B	0.25-2	1	1	0	100
	Colistin	0.25-2	1	1	0	100
	Amikacin	0.25-8	4	8	0	100
	Ceftazidime	0.125-64	2	8	4.3	95.7
	Cefepime	0.06-32	2	8	2.2	95.7
	Ceftazidime-avibactam	0.06-8	2	2	4.3	95.7
	Cefoperazone-sulbactam	1-64	8	16	–	–
	Meropenem	0.06-2	0.125	0.5	0	100
	Ciprofloxacin	0.06-4	0.125	0.5	6.5	91.3
carbapenem-resistant *Pseudomonas aeruginosa* (n = 54)	MRX-8	0.5->32	1	1	1.9	98.1
	Polymyxin B	0.5->32	1	1	1.9	98.1
	Colistin	0.5->8	1	1	1.9	98.1
	Amikacin	1->64	4	16	9.3	90.7
	Ceftazidime	4->64	32	>64	59.3	20.4
	Cefepime	4->64	16	64	42.6	25.9
	Ceftazidime-avibactam	2->64	8	32	72.2	27.8
	Cefoperazone-sulbactam	16->64	64	>64	–	–
	Meropenem	8->16	16	>16	100	0
	Ciprofloxacin	0.06->64	1	32	40.7	38.9

R, resistant; S, susceptible. All the intermediate data were not showed in table.

**Table 5:** *In vitro* activity of MRX-8 and other comparator agents against 113 of *Acinetobacter baumannii* (mg/L).

Isolates	Antimicorbial agents	MIC (mg/liter)			R, %	S, %
		Range	50%	90%		
carbapenem-susceptible *Acinetobacter baumannii* (n = 43)	MRX-8	0.125->32	0.5	0.5	4.7	95.3
	Polymyxin B	0.5->32	0.5	0.5	4.7	95.3
	Colistin	0.5-16	0.5	0.5	4.7	95.3
	Amikacin	0.5-16	2	4	0	100
	Ceftazidime	0.5-8	4	4	0	100
	Cefepime	0.25-4	2	4	0	100
	Ceftazidime-avibactam	0.125-8	4	4	–	–
	Cefoperazone-sulbactam	1-4-	2	4	–	–
	Meropenem	0.06-0.5	0.25	0.25	0	100
	Ciprofloxacin	0.06-64	0.125	8	14	86
carbapenem-resistant *Acinetobacter baumannii* (n = 70)	MRX-8	0.25->32	0.5	1	2.9	97.1
	Polymyxin B	0.25->32	0.5	1	2.9	97.1
	Colistin	0.25->8	0.5	1	2.9	97.1
	Amikacin	2->64	>64	>64	87.1	12.9
	Ceftazidime	64->64	>64	>64	100	0
	Cefepime	8->64	>64	>64	95.7	1.4
	Ceftazidime-avibactam	1->64	64	64	–	–
	Cefoperazone-sulbactam	8->64	>64	>64	–	–
	Meropenem	16->16	>16	>16	100	0
	Ciprofloxacin	32->64	64	>64	100	0

R, resistant; S, susceptible. All the intermediate data were not showed in table.

**Table 6:** *In vitro* activity of MRX-8 and other comparator agents against 80 of other non-fermentative bacteria (mg/L).

Isolates	Antimicorbial agents	MIC (mg/liter)			R, %	S, %
		Range	50%	90%		
Stenotrophomonas maltophilia (n = 30)	MRX-8	0.25->32	8	>32	–	–
	Polymyxin B	0.25->32	8	32	–	–
	Colistin	0.5->8	8	>8	–	–
	Amikacin	4->64	>64	>64	–	–
	Ceftazidime	4->64	64	>64	63.3	23.3
	Ceftazidime-avibactam	2->64	16	64	–	–
	Cefoperazone-sulbactam	8-64	32	64	–	–
	Meropenem	>16->16	>16	>16	–	–
	Levofloxacin	0.125-16	2	8	23.3	63.3
Burkholderia cepacia (n = 30)	MRX-8	0.25->32	>32	>32	–	–
	Polymyxin B	0.5->32	>32	>32	–	–
	Colistin	0.5->8	>8	>8	–	–
	Amikacin	1->64	>64	>64	–	–
	Ceftazidime	0.25-32	8	16	10	80
	Ceftazidime-avibactam	0.25-16	4	4	–	–
	Cefoperazone-sulbactam	2->64	64	>64	–	–
	Meropenem	≤0.06-8	4	4	0	90
	Levofloxacin	≤0.06-16	2	4	10	83.3
Alcaligenes app. (n = 20)	MRX-8	0.06->32	1	4	–	–
	Polymyxin B	0.25->32	2	4	–	–
	Colistin	0.25->8	2	4	–	–
	Amikacin	4->64	>64	>64	70	30
	Ceftazidime	1->64	8	>64	30	60
	Cefepime	4->64	32	>64	–	–
	Ceftazidime-avibactam	1->64	4	64	25	60
	Cefoperazone-sulbactam	1->64	8	>64	25	70
	Meropenem	0.06->16	0.125	>16	20	75
	Ciprofloxacin	1->64	4	64	–	–

R, resistant; S, susceptible. All the intermediate data were not showed in table.

**Table 7:** In vitro activity of MRX-8 and other comparator agents against 25 of *Haemophilus spp.*(mg/L).

Isolates	Antimicorbial agents	MIC (mg/liter)			R, %	S, %
		Range	50%	90%		
Haemophilus spp. (n = 25)	MRX-8	0.06-1	0.25	0.5	–	–
	Polymyxin B	0.125-1	0.5	1	–	–
	Ampicillin	0.125->32	4	>32	52	44
	Ampicillin-sulbactam	0.06->32	1	32	16	84
	Ceftriaxone	≤0.03->32	0.06	0.25	–	96
	Levofloxacin	≤0.03-8	≤0.03	1	–	92

R, resistant; S, susceptible. All the intermediate data were not showed in table.

### *In Vitro* Antimicrobial Activity Comparison With Comparators

For polymyxins-susceptible and carbapenem-resistant strains, the antibacterial activity of MRX-8 against *E. coli* and *K. pneumoniae* was in accordance with that of polymyxin B and colistin (100.0% susceptibility); and better than that of amikacin (56.5% and 23.3% susceptibility) and ceftazidime-avibactam (15.2% and 76.7% susceptibility); and significantly superior to other antibacterial agents (0-1.7% susceptibility). For polymyxins-susceptible and carbapenem-susceptible strains, the antibacterial activity of MRX-8 against *E. coli* and *K. pneumoniae* was in accordance with that of polymyxin B and colistin (100.0% susceptibility); and similar with amikacin (93.1% and 100% susceptibility) and ceftazidime-avibactam (91.4% and 100.0% susceptibility). For polymyxin-resistant strains, there’re still 5.6% of *E. coli* and 9.4% of *K. pneumoniae* isolates were susceptible to MRX-8 (**Tables 1**, **2**).

As for other *Enterobacterales*, the antibacterial activity of MRX-8 against other *Klebsiella* spp. and *Citrobacter* spp. strains was little worse than that of amikacin (92.0-96.0% versus 100% susceptibility), and better than other antibacterial agents. For *Morganella* spp.*, Proteus* spp.*, Providencia* spp.*, and Serratia* spp. strains, all were naturally resistant to MRX-8, polymyxin B and colistin, but most were susceptible to amikacin, ceftazidime-avibactam, cefoperazone-sulbactam, meropenem, and part of cephalosporins. For *Salmonella enterica* strains, the antibacterial activity of MRX-8 little worse than that of amikacin, ceftazidime/avibactam and meropenem (94.1% versus 100.0% susceptibility), and was similar to other antibacterial agents (82.4% to 94.1% susceptibility) except ciprofloxacin. For *Shigella* spp.strains, the antibacterial activity of MRX-8 was in accordance with that of amikacin, ceftazidime/avibactam and meropenem (100.0% susceptibility), and was superior to other antibacterial agents (50.0%-90.0% susceptibility) (**Table 3**).

For carbapenem-susceptible *P. aeruginosa* and *A. baumannii*, MRX-8 showed similar *in vitro* antibacterial activity with polymyxin B, colistin, amikacin, ceftazidime and cefepime (100.0% versus 95.7-100.0% susceptibility; 95.3% versus 95.3-100.0% susceptibility). However, for carbapenem-resistant *P. aeruginosa* and *A. baumannii*, it was significantly superior to comparators except polymyxins (**Tables 4**, **5**). The antibacterial activity of MRX-8 against *S. maltophilia* was worse than levofloxacin, and similarly poor to polymyxin B, colistin and other antibacterial agents. The antibacterial activity of MRX-8 against *B. cepacia* was inferior to that of ceftazidime, ceftazidime-avibactam, meropenem and levofloxacin, and similar to that of others. The antibacterial activity of MRX-8 against *Alcaligenes app.* was similar to that of polymyxin B and colistin, and better than other antibacterial agents (**Table 6**). The antibacterial activity of MRX-8 and polymyxin B against *Haemophilus* spp. was similarly strong to that of ampicillin and ampicillin-sulbactam, but little worse than ceftriaxone and levofloxacin (96.0% and 92.0% susceptibility) (**Table 7**).

## Disscussion

At present, the clinical use of polymyxins antibiotics in China are mainly two elements, polymyxin B and colistin. Polymyxins play an antibacterial role mainly by interacting with lipopolysaccharides (LPS) lipid A component of bacterial cell membrane to increase the permeability of bacterial outer membrane (Vaara, 1992). Therefore, one of the main mechanisms of bacterial resistance to polymyxins is the modification of LPS in outer membrane. Plasmid-mediated colistin resistance gene *mcr-1* was first discovered in *E. coli* origined from animals in 2015, and this gene could transmit between different species through plasmids, mediating low levels of colistin resistance (Liu et al., 2016). Currently, mcr-1 to mcr-9 subtypes of mcr genes has been reported worldwide, and the most common one in China is mcr-1, followed by mcr-3 and mcr-8, which are mainly distributed in *Enterobacterales* with low detection rates (0.52% to 1.62%) (Shi et al., 2020). In 2018, Results from the China antimicrobial resistance surveillance network (CHINET) of 2018 showed that 87.9% and 93.8% of the carbapenem-resistant *E. coli* and *K. pneumoniae* were susceptible to polymyxin B, suggesting that polymyxin B still has high antibacterial activity against the multi-drug-resistant strains, especially carbapenem-resistant strains (Yang et al., 2020). As one of the optional “last line of defense” antibiotics, polymyxins are often clinically used to treat the infection of multidrug-resistant gram-negative bacteria.

MRX-8 is a new generation of polymyxins, containing a fatty acyl tail attached *via* an ester bond, which allows for deesterification to a less toxic metabolite (Duncan, 2022). After modification, not only the antibacterial activity is enhanced, but also the renal toxicity is significantly reduced compared to polymyxin B (Duncan, 2022). Seen from this study, the MICs of polymyxin B and colistin was 2-4 times higher than that of MRX-8 when against *E. coli, Citrobacter* spp. and *Shigella* spp., and was 1-2 times higher when against *Alcaligenes app.*, *K. pneumoniae* and *Haemophilus* spp., and the same as that of MRX-8 when against *P. aeruginosa* or *A. baumannii*, indicating that the antibacterial activity of MRX-8 against most tested bacteria was slightly better than or similar to that of polymyxin B and colistin. We also found that when strains were resistant to polymyxin B or colistin, most of them were also resistant to MRX-8 (94.4% of *E. coli* and 91.6% of *K. pneumoniae*), so we could speculate that *in vitro* activity of MRX-8 is little excellent than polymyxin B or colistin. Considering the markedly reduced nephrotoxicity of MRX-8 over PMB has been confirmed *in vivo*, MRX-8 is potential for improved efficacy against certain Gram-infections (Duncan, 2022).

For CR-PAE, MRX-8, polymyxin B, colistin and amikacin all showed excellent antibacterial activity, which was far better than that of ceftazidime-avibactam. For CR-ABA, except MRX-8, polymyxin B and colistin showed good antibacterial activity, the other drugs all showed poor antibacterial activity. Currently, there are few *in vitro* pharmacodynamics studies of MRX-8 either at home and abroad. Lepak AJ et al. compared the *in vivo* antimicrobial activity of MRX-8 and colistin using the thigh and lung infection models of granulocytopenia mice, showing that MRX-8 and colistin had good *in vivo* antibacterial activity against *E. coli, K. pneumoniae, P. aeruginosa* and *A. baumannii*, and the activity increased with the increase of dose (Lepak et al., 2020).

One of the limitation of this study is the number of strains of each bacteria is small. In the future, continuous *in vitro* antibacterial activity studies are needed to monitor the resistant change of bacteria to MRX-8.

## Conclusion

MRX-8 was more effective against most clinically isolated *Enterobacteriaceae*, including carbapenem-resistant *E. coli* and *K. pneumoniae*, highlighting its potential as valuable therapeutics.

## Data Availability Statement

The raw data supporting the conclusions of this article will be made available by the authors, without undue reservation.

## Ethics Statement

This study was granted exemption from requiring ethics approval, because none of animals or human samples were involved in this study and no potentially identifiable human images or data was presented in this manuscript.

## Author Contributions

All authors contributed to the experiment operation and data collection in this study. FH contributed to the study conception and design. Material preparation and analysis were mainly performed by SW and PZ. The first draft of the manuscript was written by DY and FH revised on previous versions of the manuscript. All authors read and approved the final manuscript.

## Funding

This work was funded by the National Key Research and Development Program of China (2021YFC2701803), the China Antimicrobial Surveillance Network (Independent Medical Grants from Pfizer, 2018QD100), Shanghai MicuRx Pharmaceutical Co. Ltd and Shanghai Antimicrobial. Surveillance Network (3030231003). The funders had no role in study design, data collection and analysis, decision to publish or preparation of the manuscript.

## Conflict of Interest

The authors declare that the research was conducted in the absence of any commercial or financial relationships that could be construed as a potential conflict of interest.

## Publisher’s Note

All claims expressed in this article are solely those of the authors and do not necessarily represent those of their affiliated organizations, or those of the publisher, the editors and the reviewers. Any product that may be evaluated in this article, or claim that may be made by its manufacturer, is not guaranteed or endorsed by the publisher.
